# PD-L1 as a Urine Biomarker in Renal Cell Carcinoma—A Case Series and Proof-of-Concept Study

**DOI:** 10.3390/diagnostics14070741

**Published:** 2024-03-30

**Authors:** Philipp Reimold, Georgi Tosev, Adam Kaczorowski, Jana Friedhoff, Constantin Schwab, Viktoria Schütz, Magdalena Görtz, Niklas Panzer, Martina Heller, Cem Aksoy, Ruth Himmelsbach, Thomas Walle, Stefanie Zschäbitz, Dirk Jäger, Anette Duensing, Albrecht Stenzinger, Markus Hohenfellner, Stefan Duensing

**Affiliations:** 1 Department of Urology, University Hospital Heidelberg, Im Neuenheimer Feld 420, D-69120 Heidelberg, Germany; 2 Molecular Urooncology, University Hospital Heidelberg, Im Neuenheimer Feld 517, D-69120 Heidelberg, Germany; 3 Institute of Pathology, University Hospital Heidelberg, Im Neuenheimer Feld 224, D-69120 Heidelberg, Germany; 4 Department of Medical Oncology, University Hospital Heidelberg and National Center for Tumor Diseases (NCT), Im Neuenheimer Feld 460, D-69120 Heidelberg, Germany; 5 Precision Oncology of Urological Malignancies, University Hospital Heidelberg, Im Neuenheimer Feld 517, D-69120 Heidelberg, Germany

**Keywords:** RCC, PD-L1, urine, biomarker, immunotherapy

## Abstract

Background: Renal cell carcinoma (RCC) is among the most lethal urologic malignancies once metastatic. Current treatment approaches for metastatic RCC (mRCC) involve immune checkpoint inhibitors (ICIs) that target the PD-L1/PD-1 axis. High PD-L1 expression in tumor tissue has been identified as a negative prognostic factor in RCC. However, the role of PD-L1 as a liquid biomarker has not yet been fully explored. Herein, we analyze urine levels of PD-L1 in mRCC patients before and after either ICI therapy or surgical intervention, as well as in a series of patients with treatment-naïve RCC. Patients and Methods: The mid-stream urine of patients with mRCC (*n* = 4) or treatment-naïve RCC, i.e., prior to surgery from two centers (cohort I, *n* = 49: cohort II, *n* = 29) was analyzed for PD-L1 by ELISA. The results from cohort I were compared to a control group consisting of patients treated for non-malignant urologic diseases (*n* = 31). In the mRCC group, urine PD-L1 levels were measured before and after tumor nephrectomy (*n* = 1) or before and after ICI therapy (*n* = 3). Exosomal PD-L1 in the urine was analyzed in selected patients by immunoblotting. Results: A strong decrease in urine PD-L1 levels was found after tumor nephrectomy or following systemic treatment with ICIs. In patients with treatment-naïve RCC (cohort I), urine PD-L1 levels were significantly elevated in the RCC group in comparison to the control group (median 59 pg/mL vs. 25.7 pg/mL, *p* = 0.011). PD-L1 urine levels were found to be elevated, in particular, in low-grade RCCs in cohorts I and II. Exosomal PD-L1 was detected in the urine of a subset of patients. Conclusion: In this proof-of-concept study, we show that PD-L1 can be detected in the urine of RCC patients. Urine PD-L1 levels were found to correlate with the treatment response in mRCC patients and were significantly elevated in treatment-naïve RCC patients.

## 1. Introduction

Renal cell carcinoma (RCC) represents the majority of kidney tumors and ranks among the top ten cancers in both men and women [1,2]. Once metastatic, RCC belongs to the most lethal urologic malignancies [3]. There are currently no routinely used tools for screening, and most patients are diagnosed incidentally, i.e., typically by an abdominal ultrasound [4].

Therapeutic options in RCC range from the surveillance of small renal masses, nephron-sparing surgery for resectable RCC, extended surgery for locally advanced tumors and immunotherapy alone or in combination with antiangiogenetic agents in patients with metastatic RCC (mRCC) [5,6,7,8].

Drugs targeting the programmed death-1 (PD-1)/PD-ligand 1 (PD-L1) axis are currently proposed by EAU guidelines as first- as well as subsequent therapies for mRCC patients in all risk groups when no contraindication for an immune checkpoint blockade exists [9].

PD-L1 is a transmembrane protein that is expressed on immune cells, including antigen-presenting cells and was found to be overexpressed in various cancer cells [10]. When bound to PD-1, PD-L1 promotes malignant progression by allowing the immune evasion of tumor cells [11].

Although PD-L1 blockade is widely used therapeutically, the role of PD-L1 as a prognostic biomarker is not as clear in RCC when compared to other malignancies, e.g., bladder cancer. In urothelial carcinoma of the bladder, histopathologic scores such as the IC-score or CPS-score correlate with the treatment response to PD-L1 inhibitors [12,13]. In mRCC, the available evidence is not as conclusive. The enhanced expression of PD-L1 in tumor tissue seems to be a predictive marker for improved progression-free survival (PFS), however, without affecting the overall survival (OS) [14]. As the definition of PD-L1 positivity in mRCC is somewhat inconsistent [15], further studies on the role of PD-L1 as a biomarker in RCC are warranted. In particular, the potential role of PD-L1 as a liquid biomarker in blood or urine has not been sufficiently explored. The feasibility of detecting PD-L1 in urine samples of bladder cancer patients has recently been shown by our group [16]. Urine PD-L1 expression may also reflect PD-L1 signaling outside the tumor (e.g., on dendritic cells) [17,18,19].

In this proof-of-concept study, we report the detection of PD-L1 in the urine of RCC patients. We show that urine PD-L1 levels correlate with the response to surgery or immune checkpoint blockade in mRCC patients. Furthermore, baseline PD-L1 urine levels were significantly elevated in treatment-naïve RCC patients.

## 2. Patients and Methods

### 2.1. Patients

Mid-stream urine samples were collected at the Department of Urology of the University Hospital Heidelberg, the National Center for Tumor Diseases (NCT) Heidelberg (cohort I) and the Department of Urology of the Philipps-University Marburg (cohort II). Longitudinal urine samples from four patients with mRCC were collected at the Department of Urology Heidelberg and the NCT Heidelberg.

Patients of cohort I (University Hospital Heidelberg) presented either with a renal mass (Table 1) or non-malignant urologic diseases. Urine was collected before surgery for the renal mass, and patients were subsequently assigned either to the RCC group (*n* = 49) or to the control group according to the final pathology report. Hence, the control group consisted of patients in which an RCC was not confirmed pathologically as well as patients presenting with non-malignant urologic diseases (*n* = 31; Table 1 and Table 2).

Urine samples from patients of cohort II (Philipps-University Marburg) were likewise collected prior to surgery and analyzed only when an RCC was diagnosed (Table 3).

For the analysis of PD-L1 levels in mRCC patients, urine samples were collected before and after treatment with either immunotherapy or surgery. 

Written informed consent was obtained from all participants of the study. All reported investigations were conducted according to the updated version of the Declaration of Helsinki. The study was approved by the Ethics Committee of the University of Heidelberg School of Medicine (vote 206/2005, vote 207/2005, vote S-864/2019) as well as the Ethics Committee of the Philipps-University Marburg (Az.: 24-44-BO).

### 2.2. Urine Samples and PD-L1 Analysis

All urine samples were stored at room temperature using urine collection and preservation tubes from Norgen Biotek (Thorold, ON, Canada, catalog #318122). Whole urine without centrifugation was used, and tumor and control samples were stored for the same length of time. A Quantikine^®^ ELISA for Human/Cynomolgus Monkey PD-L1/B7-H1 from R&D Systems (Minneapolis, MN, USA, catalog #DB7H10) was used to measure PD-L1 in the urine samples. In total, 100 μL of urine was used, and experiments were performed in duplicates. Assay performance was tested by creating a standard curve for each experiment, and only experiments with a linear curve were considered for further analysis. 

### 2.3. Immunoblot Analysis of Exosomes

Exosomes were extracted with the Total Exosome Extraction Reagent (Invitrogen, Carlsbad, CA, USA) from urine samples. Proteins were isolated from exosomes using the Total Exosome RNA and Protein Isolation Kit (Invitrogen). For immunoblot analysis, 10 µL of protein lysates were separated by SDS-PAGE and blotted onto nitrocellulose membranes. The membranes were incubated with primary antibodies directed against TSG101 (Novus Biologicals, Centennial, CO, USA) or PD-L1 (GeneTex, Irvine, CA, USA) at 4 °C overnight. After incubation with horseradish peroxidase-conjugated secondary antibodies (Invitrogen), proteins were detected with the ImageQuant™ LAS 4000 mini (GE HealthCare, Chicago, IL, USA) system using the Pierce™ ECL Western Blotting substrate (Thermo Scientific, Waltham, MA, USA). All primary antibodies were used at a 1:1000 dilution.

### 2.4. Targeted Next Generation Sequencing (NGS)

The targeted NGS of tumor tissue from selected patients was performed using the TruSight Oncology 500 panel (Illumina, San Diego, CA, USA), as previously described [20,21].

### 2.5. Statistical Analysis

Statistical analysis was performed using SPSS^®^ Statistics for Windows, Version 23.0 (IBM^®^, Armonk, NY, USA). Statistical significance was assessed using the Mann–Whitney U test with a *p*-value of ≤0.05 considered significant. 

## 3. Results

### 3.1. Urine PD-L1 Levels Decrease in Patients with mRCC after Cytoreductive Nephrectomy or Immune Checkpoint Blockade

Case study 1:

A 58-year-old woman (Patient 1) presented at the Department of Urology of the University Hospital Heidelberg with synchronous oligometastatic clear cell RCC, including symptomatic metastases to the spine. Imaging revealed a pathologic fracture of a lumbar vertebra. A cytoreductive nephrectomy was performed, followed by the radiotherapy of the symptomatic lumbar lesion with a cumulative dose of 39 Gy.

The panel NGS of the primary tumor revealed pathogenic mutations in *PBRM1* and *PTEN* and likely pathogenic mutations in *VHL* as well as *PIK3C2G*.

**Figure 1:**
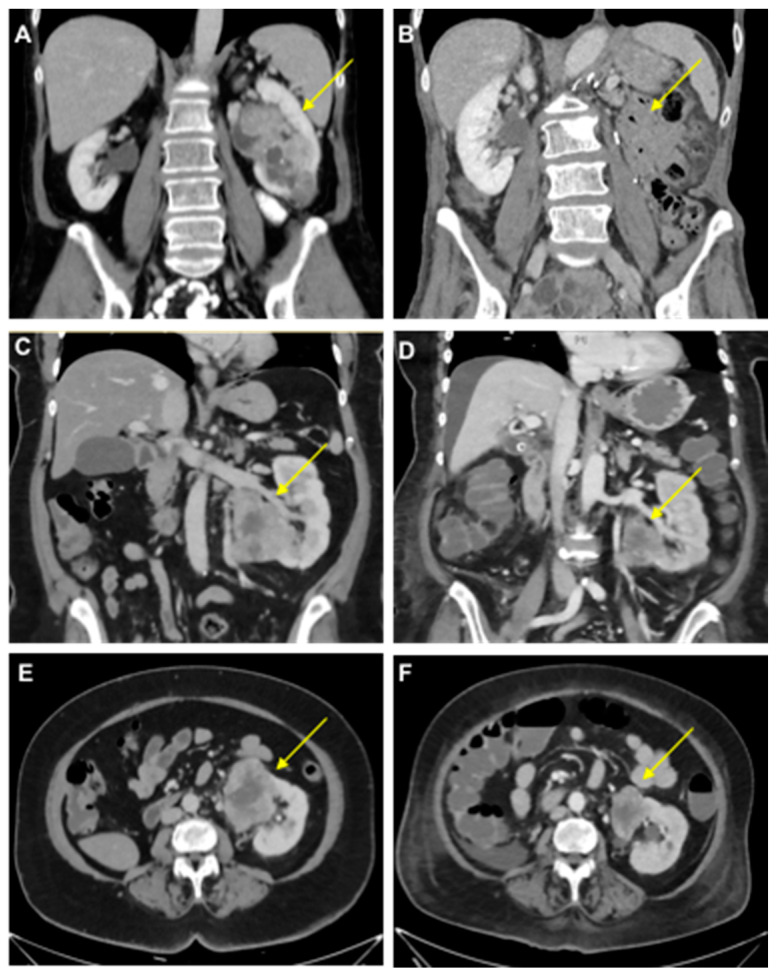
Clinical course of mRCC patients. (**A**,**B**) Computed tomography scans of the primary tumor in a 58-year-old female with oligometastatic mRCC before (**A**) and after (**B**) a left-sided tumor nephrectomy (Patient 1). (**C**–**F**) Tumor-bearing left single kidney in a 63-year-old female patient with congenital renal agenesia on the right side before (**C**,**E**) and after (**D**,**F**) the first cycle of therapy with ipilimumab/nivolumab with tumor regression (−31% after RECIST; Patient 2). Arrows indicate RCCs except in B.

**Figure 2:**
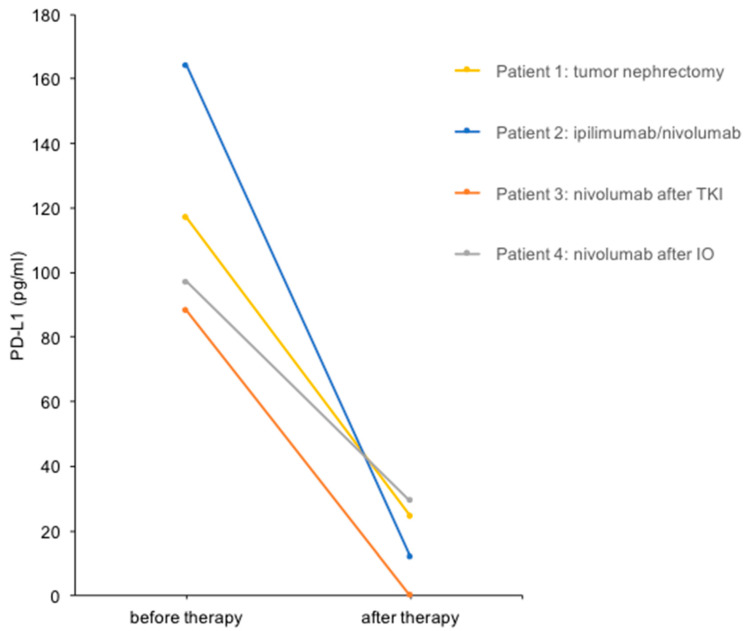
Urine PD-L1 levels before and after treatment in four patients with mRCC. Urine PD-L1 levels before and after the therapeutic interventions indicated are shown.

We performed a urine PD-L1 analysis before and after cytoreductive tumor nephrectomy before the start of radiotherapy (Figure 1). The PD-L1 level decreased from 117 pg/mL before therapy to 25 pg/mL (4.7-fold reduction) in this patient (Figure 2).

Case study 2:

A 63-year-old woman (Patient 2) presented with metastatic clear cell RCC, initially diagnosed after the resection of a frontal skull metastasis. Staging revealed a large renal tumor of the left kidney with metastatic lesions in the bones, lungs, brain, adrenal gland, lymph nodes and skull. The patient had a single kidney due to congenital renal agenesia of the right side. After resection of the skull metastasis, systemic therapy with ipilimumab and nivolumab was initiated, accompanied by local radiotherapy. The panel NGS of the primary tumor revealed pathogenic mutations in *VHL*, *BAP1* and *NF2* and likely pathogenic mutations in *KDM6A,* as well as *ABL*. After the first cycle of immunotherapy, the patient showed radiographic tumor regression (−31% after RECIST; Figure 1). The patient deceased due to complications of severe candida sepsis before the second cycle could be administered. The urine of this patient was collected and analyzed for PD-L1 before and after immunotherapy. Three weeks after the first dose of the first cycle, the urine PD-L1 level had decreased from 164 pg/mL before therapy to 12 pg/mL (13.7-fold reduction; Figure 2).

Case study 3:

A 70-year-old man (Patient 3) had received multiple systemic treatments for metastatic clear cell RCC since 2004. After cytoreductive nephrectomy (2004), he was administered IL-2/IFN-α + 5-FU (2004), sunitinib (2012–2015), pazopanib (2015–2020) and underwent surgery for metastases to the pancreas (2006), brain (2012) and lung (2017). Imaging showed progressive disease with new liver, cutaneous and adrenal metastases under therapy with pazopanib. Immunotherapy with nivolumab was initiated, and urine PD-L1 levels were measured before and after the first cycle. The PD-L1 level decreased from 88 pg/mL before nivolumab to undetectable after the first cycle in this patient (Figure 2). Follow-up imaging showed stable disease until 13 months after the initiation of nivolumab. After radiotherapy of a cutaneous metastasis and resection of another cutaneous metastasis, therapy was changed to cabozantinib after a total of 28 months on immune checkpoint inhibitors (ICIs).

Case study 4:

An 86-year-old woman (Patient 4) was diagnosed with clear cell RCC and synchronous metastases to the bones and lymph nodes. After heminephrectomy, the patient received three cycles of ipilimumab and nivolumab and showed stable disease. Due to a therapy-related autoimmune hypophysitis, the therapy regimen was changed to nivolumab monotherapy. The PD-L1 level decreased from 97 pg/mL before nivolumab treatment to 29 pg/mL (3.3-fold reduction; Figure 2). Follow-up imaging showed stable disease, and therapy was continued for 17 months until the patient passed away.

Taken together, these findings suggest a correlation between urine PD-L1 levels and disease activity in mRCC patients. 

### 3.2. Elevated Urine PD-L1 Levels in Treatment-Naïve Patients with RCC 

**Figure 3:**
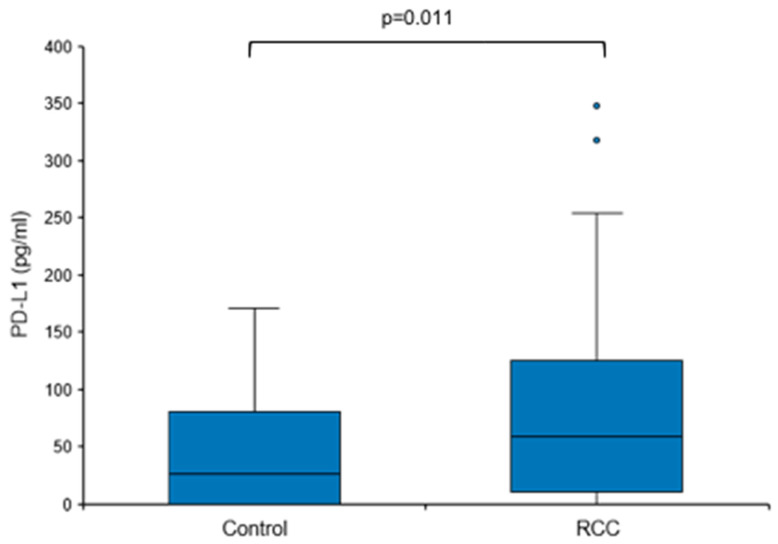
Urine PD-L1 levels are elevated in treatment-naïve RCC patients. Box plot of urine PD-L1 levels in RCC patients in comparison to a control group (Table 1 and Table 2). Statistical significance was assessed using the Mann–Whitney U test.

Based on these findings, we next sought to address the question of whether baseline urine PD-L1 levels were also elevated in treatment-naïve patients with RCC, i.e., prior to surgery or any other therapy. Urine PD-L1 was measured in patients with a renal mass suspicious for RCC before diagnosis and a control group consisting of urologic patients without malignant diseases. Only patients with histologically confirmed RCC remained assigned to the RCC group (*n* = 49); patients with no histological evidence for RCC (e.g., oncocytoma) were moved to the control group (total *n* = 31; Table 1 and Table 2). The overall urine PD-L1 detection rate was 80% (71% in the control group and 84% in the RCC group). Urine PD-L1 levels were significantly higher in the RCC group when compared to the control group (median 59.04 vs. 25.71 pg/mL, *p* = 0.011; Figure 3). 

**Figure 4:**
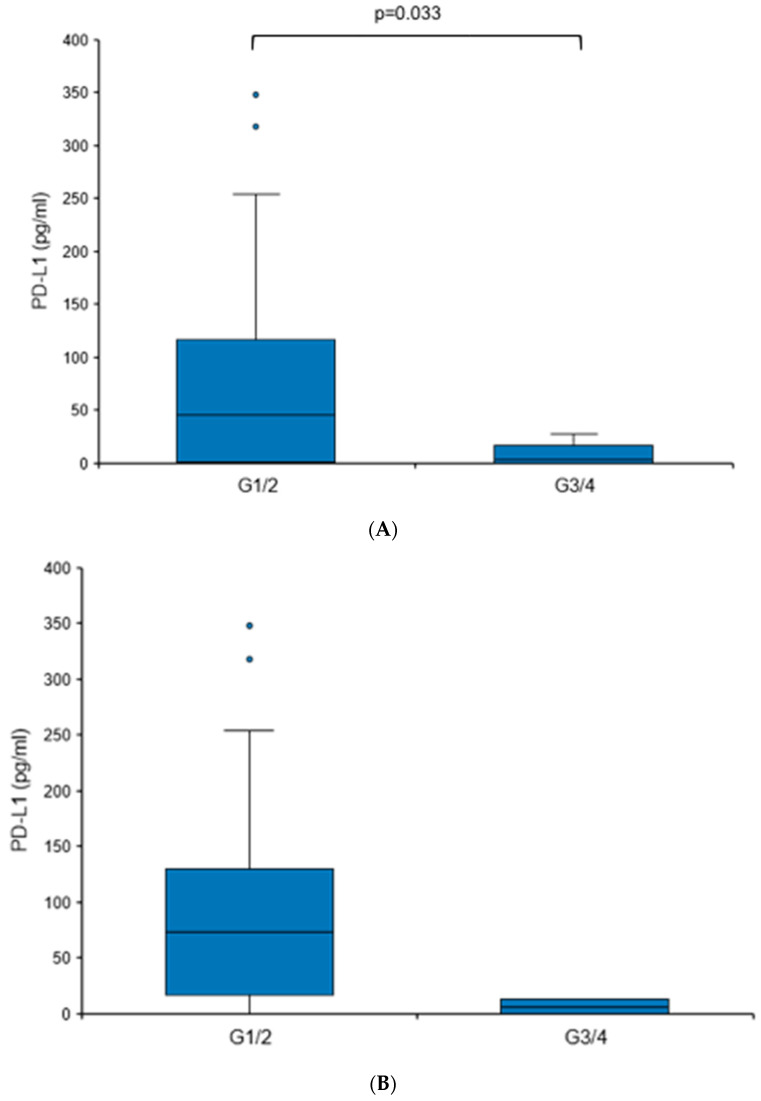
Urine PD-L1 levels are elevated in patients with low-grade RCC. Box plot of urine PD-L1 levels in RCC patients with grade 1/2 tumors in comparison to patients with grade 3/4 tumors. (**A**) Cohort I; (**B**) cohort II. Statistical significance was assessed using the Mann–Whitney U test.

When comparing tumors with different grades (G1/G2 vs. G3/G4), a statistically significant elevation in urine PD-L1 levels was found in more differentiated tumors (73.62 vs. 6.13 pg/mL, *p* = 0.033), although it needs to be mentioned that G3/G4 tumors comprised only 6.1% of the RCC cohort (Figure 4A). There were no significant differences in the urine PD-L1 levels when patients were sub-stratified according to histology (clear cell versus non-clear cell RCC), T stage, nodal status or the presence of distant metastasis.

To further corroborate the notion that PD-L1 levels may be elevated in patients with RCC showing less aggressive features, we analyzed an independent second cohort (Table 3). A similar trend was detected, albeit without reaching statistical significance (Figure 4B). The overall urine PD-L1 detection rate in cohort II was 31%. Like cohort I, there was no statistically significant correlation between PD-L1 levels and histology or T stage. 

Altogether, these results show that urine PD-L1 levels are significantly increased in treatment-naïve patients with histologically proven RCC, and in particular, low-grade tumors.

### 3.3. Exosomes May Be the Source of Urine PD-L1 in a Subset of Patients

**Figure 5:**
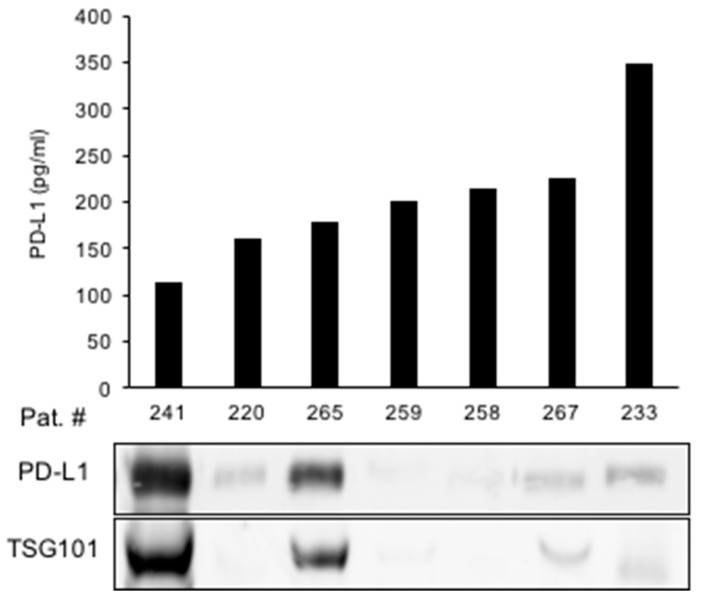
Exosomes may be a source of urine PD-L1 in a subset of RCC patients. Urine PD-L1 levels from selected patients are shown in correlation to an immunoblot analysis of exosomal PD-L1. TSG101 is shown as a marker for exosomes.

We next aimed to identify the origin of urine PD-L1 in greater detail. To this end, we tested the hypothesis that PD-L1 may be released into the urine via exosomes. Exosomes were isolated from the urine of patients with the highest PD-L1 concentrations of cohort I (Figure 5). An immunoblot analysis of extracted exosomal proteins showed a high PD-L1 expression in two samples with high expression of the exosome marker TSG101, suggesting exosomes as a source of urine PD-L1. However, PD-L1 was also detected in urine samples in which no exosomes were present (Figure 5). 

In conclusion, exosomes appear to be one, although not exclusive, vehicle for the release of PD-L1 into the urine. 

## 4. Discussion

PD-L1 is a major target for the systemic treatment of mRCC patients with ICIs. Several studies have shown that a high PD-L1 expression in tumor tissue is associated with poor patient outcomes [22,23]. The role of PD-L1 as a liquid biomarker, however, is insufficiently explored [14]. 

In this proof-of-concept study, we aimed to elucidate a possible role of PD-L1 as a liquid biomarker in the urine of patients with advanced RCC undergoing treatment as well as in treatment-naïve, i.e., pre-surgical, RCC patients. We showed that urine PD-L1 can be detected in patients with mRCC and, furthermore, that the PD-L1 levels correlate with disease activity. Urine PD-L1 levels were also found to be significantly elevated in patients with treatment-naïve RCC. We detected higher PD-L1 levels in patients with low-grade tumors, a finding that was confirmed in a second, independent patient cohort. Lastly, our results suggest that exosomes are a source of urine PD-L1 in a subset of patients. 

Several studies have previously explored PD-L1 as a blood biomarker in RCC [24,25,26,27,28]. Davidsson et al. [29] showed that urine PD-L1 can also be detected, albeit in only approximately 20% of RCC patients analyzed. This detection rate was lower than the detection rates in our cohorts (80% and 31%, respectively). It is noteworthy that our study shows higher levels of urine PD-L1 in histologically low-grade (grade 1/2) RCCs. This finding contrasts with a previous report showing higher levels of PD-L1 expression in the tissue from high-grade tumors [30]. Although a similar trend appears in bladder cancer [31], there are clearly exceptions to this notion, such as low-grade gliomas [32]. The most important difference between these studies and the present report is that urine PD-L1 was correlated to a histological tumor grade. Extracellular PD-L1 may have distinct functions compared to cellular PD-L1 during tumor progression [17,18,19], which is a notion that clearly requires further experimental proof. 

Furthermore, urine PD-L1 has been reported in the context of non-malignant renal conditions such as acute kidney injury or nephrotic syndrome [33,34,35].

Urine PD-L1 has previously also been reported in other urologic malignancies, for example, urothelial carcinoma of the bladder [16]. The prognostic potential as a liquid biomarker in this tumor entity is currently not completely understood. Notably, high amounts of PD-L1-expressing CD4^+^ T-cells were detected in the urine of patients with non-muscle invasive bladder cancer (NMIBC) under local immunotherapy with BCG and were associated with rapid recurrence after BCG treatment [36]. 

In the present study, we show that the source of urine PD-L1 may be exosomes, at least in a subset of patients. Exosomal PD-L1 has been described before, and elevated levels of exosomal PD-L1 are associated with tumor progression and negatively correlate with patient survival in various tumor entities [37]. Although exosomal PD-L1 in the blood of patients has been thoroughly described [30], data on exosomal PD-L1 in the urine of patients with RCC are missing (PubMed search terms “exosome PD-L1 urine renal carcinoma” on 14 March 2024). 

Alternative sources of urine PD-L1 could be tumor and/or immune cells. We tested this possibility, but neither tumor nor immune cells were detected in the urine cytology obtained from seven patients with therapy-naïve RCC. Soluble PD-L1 (sPD-L1), a truncated and secreted form of PD-L1, might be another source of urine PD-L1. Elevated levels of sPD-L1 in the blood have been associated with a worse prognosis in RCC [24]. In urine, the detection of soluble PD-L1 has been described in bladder cancer [38].

Limitations to our study are the relatively small number of patients analyzed, the lack of more longitudinal data and the absence of healthy individuals in the control group. Furthermore, the ELISA assay used was research-grade and has not been tested for clinical use.

In terms of translational relevance, future studies are needed to further evaluate urine PD-L1 as a tool to aid the diagnosis and screening of RCC. We believe that using urine PD-L1 as a marker of treatment response may be particularly promising, but clearly, more patients and a higher data density are required to support this notion. 

In conclusion, the present proof-of-concept study provides a basis for a further evaluation of urine PD-L1 as a liquid biomarker in RCC.

## Figures and Tables

**Table 1:** Patient characteristics (cohort I, *n* = 80).

Variable	RCC (*n* = 49)	Control (*n* = 31)
Age (years)		
Median	64	61
IQR	57–72	42–69
Sex, *n* (%)		
Female	16 (32.7)	11 (35.5)
Male	33 (67.3)	20 (64.5)
Histology, *n* (%)		
Clear cell	35 (71.4)	n.a.
Papillary	7 (14.3)	n.a.
Chromophobe	4 (8.2)	n.a.
Other	3 (6.1)	n.a.
T stage, *n* (%)		
T1	28 (57.2)	n.a.
T2	3 (6.1)	n.a.
T3	14 (28.5)	n.a.
T4	4 (8.2)	n.a.
N stage, *n* (%)		
N0	19 (38.8)	n.a.
Nx	24 (49.0)	n.a.
N1	6 (12.2)	n.a.
M stage, *n* (%)		
M0	9 (18.4)	n.a.
Mx	33 (67.3)	n.a.
M1	7 (14.3)	
Grade, *n* (%)		
G1	5 (10.2)	n.a.
G2	33 (67.4)	n.a.
G3	1 (2.0)	n.a.
G4	2 (4.1)	n.a.
Gx	8 (16.3)	n.a.

n.a., not applicable.

**Table 2:** Diagnoses of the control group (*n* = 31).

Diagnosis	*n* (%)
Angiomyolipoma	4 (12.9)
Appendicitis	1 (3.2)
Bosniak II cyst	1 (3.2)
Prostate hyperplasia	2 (6.5)
Cystitis	5 (16.1)
Epididymitis	1 (3.2)
Urolithiasis	5 (16.1)
Oncocytoma	7 (22.6)
Phimosis	1 (3.2)
Prostatitis	1 (3.2)
Pyelonephritis	2 (6.5)
Urethral stricture	1 (3.2)

**Table 3:** Patient characteristics (cohort II; *n* = 29).

Variable	RCC (*n* = 29)
Age (years)	
Median	68
IQR	62–77
Sex, *n* (%)	
Female	5 (17.2)
Male	24 (82.8)
Histology, *n* (%)	
Clear cell	19 (65.5)
Papillary	6 (20.7)
Chromophobe	2 (6.9)
Other	2 (6.9)
T stage, *n* (%)	
T1	22 (75.9)
T2	2 (6.9)
T3	5 (17.2)
T4	0 (0)
N stage, *n* (%)	
N0	18 (62.1)
Nx	10 (34.5)
N1	1 (3.4)
M stage, *n* (%)	
M0	18 (62.1)
Mx	8 (27.6)
M1	3 (10.3)
Grade, *n* (%)	
G1	6 (20.7)
G2	13 (44.8)
G3	4 (13.8)
G4	2 (6.9)
Gx	4 (13.8)

## Data Availability

The data presented in this study are available within the article.

## References

[1] Motzer R.J., Jonasch E., Agarwal N., Bhayani S., Bro W.P., Chang S.S., Choueiri T.K., Costello B.A., Derweesh I.H., Fishman M. (2017). Kidney Cancer, Version 2.2017, NCCN Clinical Practice Guidelines in Oncology. J. Natl. Compr. Cancer Netw..

[2] (2021). Leading Sites of New Cancer Cases and Deaths—2021 Estimates: Cancer Facts & Figures. https://www.cancer.org/content/dam/cancer-org/research/cancer-facts-and-statistics/annual-cancer-facts-and-figures/2021/leading-sites-of-new-cancer-cases-and-deaths.pdf.

[3] Siegel R.L., Miller K.D., Jemal A. (2020). Cancer statistics, 2020. CA Cancer J. Clin..

[4] Capitanio U., Montorsi F. (2016). Renal cancer. Lancet.

[5] Wehle M.J., Thiel D.D., Petrou S.P., Young P.R., Frank I., Karsteadt N. (2004). Conservative management of incidental contrast-enhancing renal masses as safe alternative to invasive therapy. Urology.

[6] Krabbe L.-M., Bagrodia A., Margulis V., Wood C.G. (2014). Surgical management of renal cell carcinoma. Semin. Interv. Radiol..

[7] Bedke J., Gauler T., Grünwald V., Hegele A., Herrmann E., Hinz S., Janssen J., Schmitz S., Schostak M., Tesch H. (2017). Systemic therapy in metastatic renal cell carcinoma. World J. Urol..

[8] Rini B.I., Battle D., Figlin R.A., George D.J., Hammers H., Hutson T., Jonasch E., Joseph R.W., McDermott D.F., Motzer R.J. (2019). The society for immunotherapy of cancer consensus statement on immunotherapy for the treatment of advanced renal cell carcinoma (RCC). J. Immunother. Cancer.

[9] Ljungberg B., Albiges L., Bensalah K., Bex A., Giles R.H., Hora M., Kuczyk M.A., Lam T., Marconi L., Merseburger A.S. (2021). EAU Guidelines: Edn. presented at the EAU Annual Congress Milan.

[10] Sun C., Mezzadra R., Schumacher T.N. (2018). Regulation and Function of the PD-L1 Checkpoint. Immunity.

[11] Han Y., Liu D., Li L. (2020). PD-1/PD-L1 pathway: Current researches in cancer. Am. J. Cancer Res..

[12] Eckstein M., Erben P., Kriegmair M.C., Worst T.S., Weiß C.-A., Wirtz R.M., Wach S., Stoehr R., Sikic D., Geppert C.I. (2019). Performance of the Food and Drug Administration/EMA-approved programmed cell death ligand-1 assays in urothelial carcinoma with emphasis on therapy stratification for first-line use of atezolizumab and pembrolizumab. Eur. J. Cancer.

[13] Eckstein M., Gupta S. (2019). New insights in predictive determinants of the tumor immune microenvironment for immune checkpoint inhibition: A never ending story?. Ann. Transl. Med..

[14] Carretero-González A., Lora D., Sobrino I.M., Sanz I.S., Bourlon M.T., Herranz U.A., Chanzá N.M., Castellano D., de Velasco G. (2020). The Value of PD-L1 Expression as Predictive Biomarker in Metastatic Renal Cell Carcinoma Patients: A Meta-Analysis of Randomized Clinical Trials. Cancers.

[15] Shen X., Zhao B. (2018). Efficacy of PD-1 or PD-L1 inhibitors and PD-L1 expression status in cancer: Meta-analysis. BMJ.

[16] Tosev G., Wahafu W., Reimold P., Damgov I., Schwab C., Aksoy C., Kaczorowski A., Stenzinger A., Nyarangi-Dix J., Hohenfellner M. (2021). Detection of PD-L1 in the urine of patients with urothelial carcinoma of the bladder. Sci. Rep..

[17] Peng Q., Qiu X., Zhang Z., Zhang S., Zhang Y., Liang Y., Guo J., Peng H., Chen M., Fu Y.-X. (2020). PD-L1 on dendritic cells attenuates T cell activation and regulates response to immune checkpoint blockade. Nat. Commun..

[18] Oh S.A., Wu D.-C., Cheung J., Navarro A., Xiong H., Cubas R., Totpal K., Chiu H., Wu Y., Comps-Agrar L. (2020). PD-L1 expression by dendritic cells is a key regulator of T-cell immunity in cancer. Nat. Cancer.

[19] Mayoux M., Roller A., Pulko V., Sammicheli S., Chen S., Sum E., Jost C., Fransen M.F., Buser R.B., Kowanetz M. (2020). Dendritic cells dictate responses to PD-L1 blockade cancer immunotherapy. Sci. Transl. Med..

[20] Kazdal D., Endris V., Allgäuer M., Kriegsmann M., Leichsenring J., Volckmar A.L., Harms A., Kirchner M., Kriegsmann K., Neumann O. (2019). Spatial and Temporal Heterogeneity of Panel-Based Tumor Mutational Burden in Pulmonary Adenocarcinoma: Separating Biology From Technical Artifacts. J. Thorac. Oncol. Off. Publ. Int. Assoc. Study Lung Cancer.

[21] Friedhoff J., Schneider F., Jurcic C., Endris V., Kirchner M., Sun A., Bolnavu I., Pohl L., Teroerde M., Kippenberger M. (2023). BAP1 and PTEN mutations shape the immunological landscape of clear cell renal cell carcinoma and reveal the intertumoral heterogeneity of T cell suppression: A proof-of-concept study. Cancer Immunol. Immunother..

[22] Denize T., Hou Y., Pignon J.-C., Walton E., West D.J., Freeman G.J., Braun D.A., Wu C.J., Gupta S., Motzer R.J. (2022). Transcriptomic Correlates of Tumor Cell PD-L1 Expression and Response to Nivolumab Monotherapy in Metastatic Clear Cell Renal Cell Carcinoma. Clin. Cancer Res. Off. J. Am. Assoc. Cancer Res..

[23] Mori K., Abufaraj M., Mostafaei H., Quhal F., Fajkovic H., Remzi M., Karakiewicz P.I., Egawa S., Schmidinger M., Shariat S.F. (2021). The Predictive Value of Programmed Death Ligand 1 in Patients with Metastatic Renal Cell Carcinoma Treated with Immune-checkpoint Inhibitors: A Systematic Review and Meta-analysis. Eur. Urol..

[24] Frigola X., Inman B.A., Lohse C.M., Krco C.J., Cheville J.C., Thompson R.H., Leibovich B., Blute M.L., Dong H., Kwon E.D. (2011). Identification of a soluble form of B7-H1 that retains immunosuppressive activity and is associated with aggressive renal cell carcinoma. Clin. Cancer Res. Off. J. Am. Assoc. Cancer Res..

[25] Kushlinskii N.E., Gershtein E.S., Morozov A.A., Goryacheva I.O., Filipenko M.L., Alferov A.A., Bezhanova S.D., Bazaev V.V., Kazantseva I.A. (2019). Soluble Ligand of the Immune Checkpoint Receptor (sPD-L1) in Blood Serum of Patients with Renal Cell Carcinoma. Bull. Exp. Biol. Med..

[26] Fukuda T., Kamai T., Masuda A., Nukui A., Abe H., Arai K., Yoshida K.I. (2016). Higher preoperative serum levels of PD-L1 and B7-H4 are associated with invasive and metastatic potential and predictable for poor response to VEGF-targeted therapy and unfavorable prognosis of renal cell carcinoma. Cancer Med..

[27] Larrinaga G., Solano-Iturri J.D., Errarte P., Unda M., Loizaga-Iriarte A., Pérez-Fernández A., Echevarría E., Asumendi A., Manini C., Angulo J.C. (2021). Soluble PD-L1 Is an Independent Prognostic Factor in Clear Cell Renal Cell Carcinoma. Cancers.

[28] Wang Q., Zhang J., Tu H., Liang D., Chang D.W., Ye Y., Wu X. (2019). Soluble immune checkpoint-related proteins as predictors of tumor recurrence, survival, and T cell phenotypes in clear cell renal cell carcinoma patients. J. Immunother. Cancer.

[29] Davidsson S., Huotilainen S., Carlsson J., Sundqvist P. (2022). Soluble Levels of CD163, PD-L1, and IL-10 in Renal Cell Carcinoma Patients. Diagnostics.

[30] Thompson R.H., Kuntz S.M., Leibovich B.C., Dong H., Lohse C.M., Webster W.S., Sengupta S., Frank I., Parker A.S., Zincke H. (2006). Tumor B7-H1 is associated with poor prognosis in renal cell carcinoma patients with long-term follow-up. Cancer Res..

[31] Kawahara T., Ishiguro Y., Ohtake S., Kato I., Ito Y., Ito H., Makiyama K., Kondo K., Miyoshi Y., Yumura Y. (2018). PD-1 and PD-L1 are more highly expressed in high-grade bladder cancer than in low-grade cases: PD-L1 might function as a mediator of stage progression in bladder cancer. BMC Urol..

[32] Martin A.M., Bell W.R., Yuan M., Harris L., Poore B., Arnold A., Engle E.L., Asnaghi L., Lim M., Raabe E.H. (2020). PD-L1 Expression in Pediatric Low-Grade Gliomas Is Independent of BRAF V600E Mutational Status. J. Neuropathol. Exp. Neurol..

[33] Wang J., Zheng X., Jiang Y., Jia H., Shi X., Han Y., Li Q., Li W. (2022). Soluble Programmed Cell Death Protein 1 and Its Ligand: Potential Biomarkers to Predict Acute Kidney Injury After Surgery in Critically Ill Patients. J. Inflamm. Res..

[34] Afaneh C., Muthukumar T., Lubetzky M., Ding R., Snopkowski C., Sharma V.K., Seshan S., Dadhania D., Schwartz J.E., Suthanthiran M. (2010). Urinary cell levels of mRNA for OX40, OX40L, PD-1, PD-L1, or PD-L2 and acute rejection of human renal allografts. Transplantation.

[35] Xuan Y., Chen F., Qin L., He R., Yuan J. (2021). sPD-1 and sPD-L1 Levels in Serum and Urine of Patients with Primary Nephrotic Syndrome and their Clinical Significance. Clin. Lab..

[36] Chevalier M.F., Schneider A.K., Cesson V., Dartiguenave F., Lucca I., Jichlinski P., Nardelli-Haefliger D., Derré L. (2018). Conventional and PD-L1-expressing Regulatory T Cells are Enriched During BCG Therapy and may Limit its Efficacy. Eur. Urol..

[37] Morrissey S.M., Yan J. (2020). Exosomal PD-L1: Roles in Tumor Progression and Immunotherapy. Trends Cancer.

[38] Vikerfors A., Davidsson S., Frey J., Jerlström T., Carlsson J. (2021). Soluble PD-L1 in Serum and Urine in Urinary Bladder Cancer Patients. Cancers.

